# Various Energetic Metabolism of Microglia in Response to Different Stimulations

**DOI:** 10.3390/molecules28114501

**Published:** 2023-06-01

**Authors:** Xiaohui Liu, Ning Jiang, Wenxia Zhou

**Affiliations:** 1 School of Traditional Chinese Medicine, Guangdong Pharmaceutical University, Guangzhou 510006, China; liuxiaohui010203@163.com; 2 Beijing Institute of Pharmacology and Toxicology, Beijing 100850, China; 3 State Key Laboratory of Toxicology and Medical Countermeasure, Beijing 100850, China

**Keywords:** Alzheimer’s disease, microglia, DAMPs, PAMPs, cytokines, energetic metabolism

## Abstract

The activation of the microglia plays an important role in the neuroinflammation induced by different stimulations associated with Alzheimer’s disease (AD). Different stimulations, such as pathogen-associated molecular patterns (PAMPs), damage-associated molecular patterns (DAMPs) and cytokines, trigger a consequence of activation in the microglia with diverse changes of the microglial cell type response in AD. The activation of the microglia is often accompanied by metabolic changes in response to PAMPs, DAMPs and cytokines in AD. Actually, we do not know the distinct differences on the energetic metabolism of microglia when subject to these stimuli. This research assessed the changes of the cell type response and energetic metabolism in mouse-derived immortalized cells (BV-2 cells) induced by a PAMP (LPS), DAMPs (Aβ and ATP) and a cytokine (IL-4) in mouse-derived immortalized cells (BV-2 cells) and whether the microglial cell type response was improved by targeting the metabolism. We uncovered that LPS, a proinflammatory stimulation of PAMPs, modified the morphology from irregular to fusiform, with stronger cell viability, fusion rates and phagocytosis in the microglia accompanied by a metabolic shift to the promotion of glycolysis and the inhibition of oxidative phosphorylation (OXPHOS). Aβ and ATP, which are two known kinds of DAMPs that trigger microglial sterile activation, induced the morphology from irregular to amoebic, and significantly decreased others in the microglia, accompanied by boosting or reducing both glycolysis and OXPHOS. Monotonous pathological changes and energetic metabolism of microglia were observed under IL-4 exposure. Further, the inhibition of glycolysis transformed the LPS-induced proinflammatory morphology and decreased the enhancement of LPS-induced cell viability, the fusion rate and phagocytosis. However, the promotion of glycolysis exerted a minimal effect on the changes of morphology, the fusion rate, cell viability and phagocytosis induced by ATP. Our study reveals that microglia induced diverse pathological changes accompanied by various changes in the energetic metabolism in response to PAMPs, DAMPs and cytokines, and it may be a potential application of targeting the cellular metabolism to interfere with the microglia-mediated pathological changes in AD.

## 1. Introduction

The progress of Alzheimer’s disease (AD) as a kind of neurodegenerative disease is characterized by cognitive impairment and an executive barrier [1]. The microglia play a pivotal role in the immune responses of the nervous system (CNS) as the resident immune cells in the brain. Activated microglia mediate the inflammatory response when subjected to different stimuli, such as pathogen-associated molecular patterns (PAMPs), damage-associated molecular patterns (DAMPs) and cytokines [2]. The microglia mediate the inflammatory response, accompanied by changes of metabolic states when subjected to PAMPs, DAMPs and cytokines, which is highly associated with AD pathophysiology [3,4,5]. PAMPs are conserved non-self and highly expressed molecular motifs that mostly have fundamentally functional components, such as lipopolysaccharide (LPS) [6]. DAMPs are a range of intracellular molecules that are not only released passively by dead cells but can also be expressed actively by live cells facing life-threatening stress, such as amyloid beta (Aβ) and intracellular adenosine 5′-triphosphate (ATP) [7,8]. Anti-inflammatory cytokines, such as interleukin-4 (IL-4), are a kind of stimuli that drive the microglia toward an anti-inflammatory phenotype.

Alterations of the microglial cell type response need a large amount of energy and microglia are required to modulate their metabolic states under different activation states [9,10]. However, little is known about the differences in the microglial energetic metabolism induced by PAMPs, DAMPs and anti-inflammatory cytokines. Previous studies mainly focus on PAMP-induced metabolic changes to support cell type responses in the microglia [11,12,13,14,15,16]. Actually, microglia in the CNS are more likely to be activated by DAMPs in AD, for the presence of the blood–brain barrier (BBB) [7]. However, it is largely unknown about how the microglial metabolic state is induced by DAMPs, such as Aβ and ATP. In addition, the metabolic changes induced by anti-inflammatory cytokines, such as IL-4, are uncertain. Therefore, the observation is that the metabolic changes in the microglia under different stimuli and interventions in the cellular metabolism of the microglia may be a potential approach for AD treatment.

In the present study, immortalized BV-2 microglial cells were used as cellular models of AD and were treated with LPS, Aβ, ATP and IL-4 to observe their phenotypic and metabolic changes, and to regulate the cellular metabolism to provide new insights into the interventions and treatments for AD.

## 2. Results

### 2.1. Effects of Different Stimulations on the Cell Viability and Fusion Rate of Microglia

**Figure 1:**
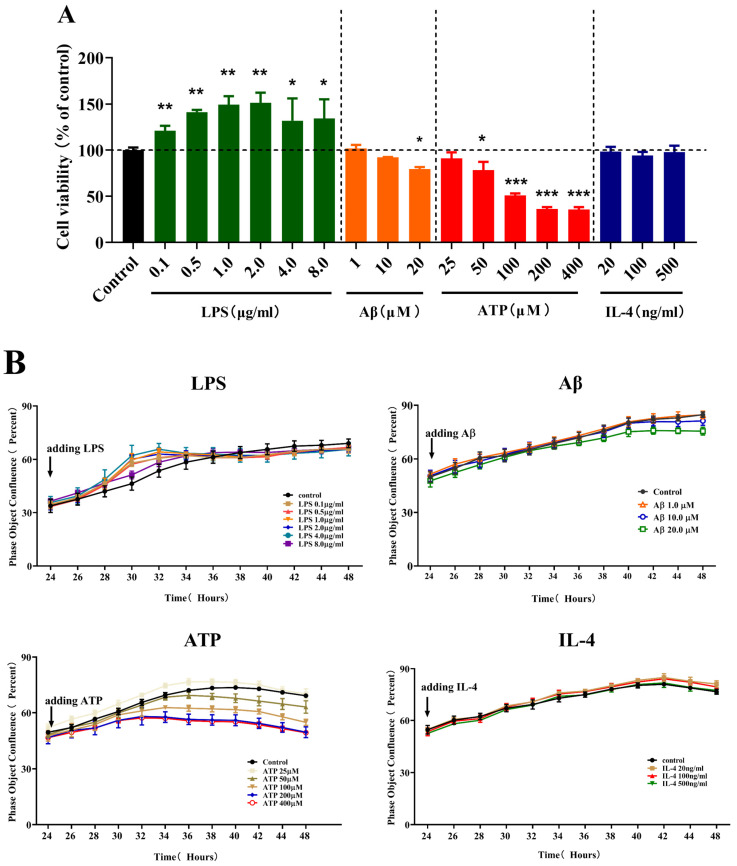
The effect of different stimulations on the cell viability and fusion rates of microglia. (**A**) The effect of different stimulations on the cell viability of microglia. One-way ANOVA with Dunnett’s multiple comparison test, * *p* < 0.05, ** *p* < 0.01, *** *p* < 0.001, compared with the control group under the same conditions. (**B**) The effect of different stimulations on the dynamic fusion rate of microglia during 24 h. BV-2 cells were treated with vehicle or 0.1, 0.5, 1.0, 2.0, 4.0 and 8.0 µg/mL LPS, 1, 10 and 20 µM Aβ, 25, 50, 100, 200 and 400 µM ATP and 20, 100, and 500 ng/mL IL-4 for 24 h. Data are presented as mean ± S.D. *n* = 3 for each experimental group. *p* < 0.05 was considered significant.

To assess the effects of different stimulations on the cell viability of microglia, we carried out a CCK-8 assay following treatment with LPS, Aβ, ATP or IL-4 for 24 h in BV-2 cells. In the presence of different stimulations, the cell viability of the microglia changed variously, as shown in Figure 1A. LPS significantly increased the cell viability in the concentration range of 0.1 (*p* < 0.01), 0.5 (*p* < 0.01), 1.0 (*p* < 0.01), 2.0 (*p* < 0.01), 4.0 (*p* < 0.05) and 8.0 µg/mL (*p* < 0.05). In addition, Aβ significantly decreased the cell viability at a concentration of 20 µM (*p* < 0.05), as well as ATP in the concentration range of 50 (*p* < 0.05), 100 (*p* < 0.001), 200 (*p* < 0.001) and 400 µM (*p* < 0.001). By contrast, IL-4 in the concentration range of 20, 100 and 500 ng/mL showed no prominent effects on the cell viability.

To determine the effects of different stimulations on the fusion rate of microglia, we used Incucyte Zoom to observe the dynamic fusion rate of the BV-2 cells during adding LPS, Aβ, ATP or IL-4 for 24 h. As shown in Figure 1B, we observed that all different stimulations had various effects on the fusion rate at 24 h. Upon LPS stimulation, the fusion rate of BV-2 cells was unchanged at different doses. Both Aβ- and ATP-treated BV-2 cells showed a reduction in the fusion rate. In the presence of Aβ, BV-2 cells showed a reduction in the fusion rate at the concentration of 20 µM (*p* < 0.01). The ATP-treated BV-2 cells showed an inhibition of fusion rate at the concentrations of 50, 100, 200 and 400 µM (*p* < 0.05, *p* < 0.001, *p* < 0.001 and *p* < 0.001). Consistent with the above observations in cell viability, IL-4 minimally affected the microglial fusion rate.

Based on the above results, we found that the cell viability and the fusion rate of microglia became stronger when treated with 1 µg/mL LPS, and these became weaker when treated with 20 µM Aβ and 100 µM ATP. Therefore, we used 1 µg/mL LPS, 20 µM Aβ and 100 µM ATP in the following experiments to observe the changes in morphology. In addition, IL-4 had no impact on the microglial cell viability and fusion rate, hence we used 100 ng/mL IL-4 in the following experiments for observing the changes of morphology, according to the previous research [17].

### 2.2. Effects of Different Stimulations on the Morphology of Microglia

**Figure 2:**
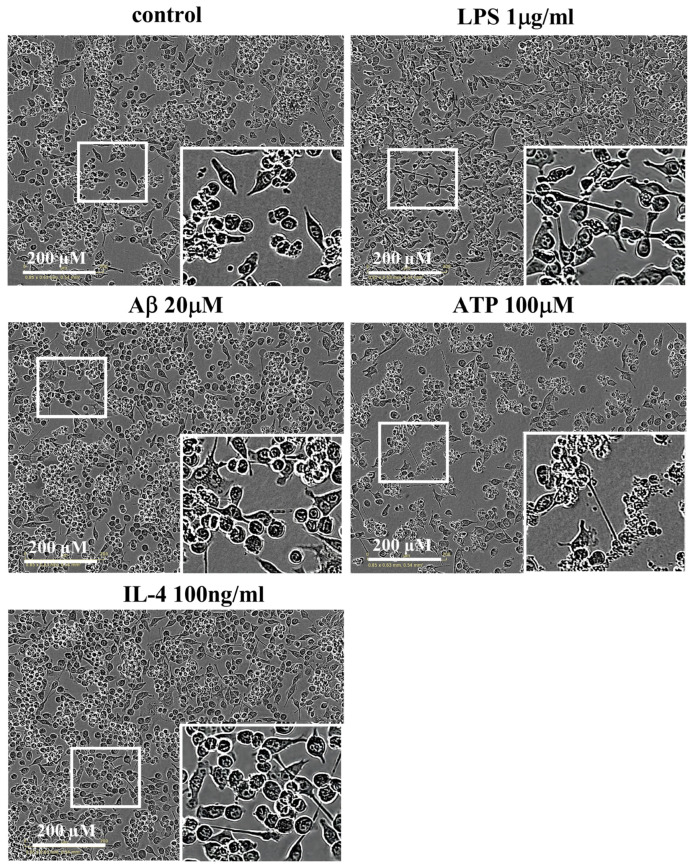
Effects of different stimulations on the morphology of the microglia. Bright-field microscopic images of BV-2 cells were exposed to vehicle, 1 µg/mL LPS, 20 µM Aβ, 100 µM ATP or 100 ng/mL IL-4 for 24 h.

To assess the effects of different stimulations on the morphology of the microglia, we used Incucyte Zoom to analyze the morphology of BV-2 cells, as shown in Figure 2. LPS-treated microglia transformed their morphology from irregular to fusiform. By contrast, the Aβ- and ATP-treated microglia transformed their morphology from irregular to amoebic, along with decreasing numbers of cells. The morphology of the microglia was minimally influenced by IL-4.

### 2.3. Effects of Different Stimulations on the Phagocytosis of Microglia

**Figure 3:**
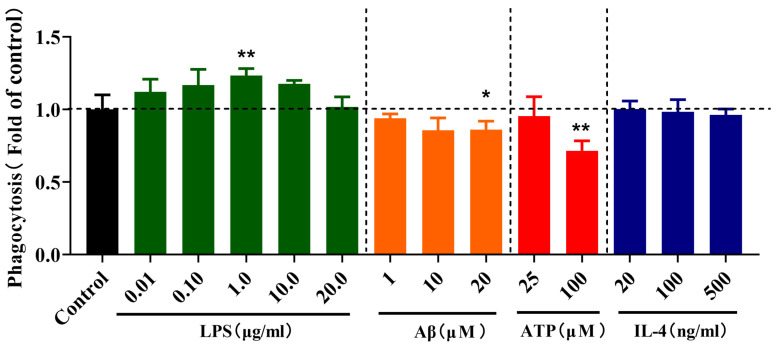
Effects of different stimulations on the phagocytosis of microglia. The BV-2 cells were treated for 24 h in vehicle controls or following treatment with 0.01, 0.1, 10.0 and 20.0 µg/mL LPS, 1, 10 and 20 µM Aβ, 25 and 100 µM ATP and 20, 100 and 500 ng/mL IL-4 in BV-2 cells for 24 h. Data are presented as mean ± S.D. * *p* < 0.05, ** *p* < 0.01, compared with the control group under the same condition; one-way ANOVA with Dunnett’s multiple comparison test for more than two groups, *n* = 3 for each experimental group. *p* < 0.05 was considered significant.

We sought to determine the effects of the different stimulations on microglial phagocytosis. As shown in the Figure 3, 1 µg/mL LPS-treated BV-2 cells exhibited a significant increase in phagocytosis (*p* < 0.01). By contrast, Aβ- (20 µM) and ATP-treated (100 µM) BV-2 cells exhibited an overall reduction (*p* < 0.05 and *p* < 0.01) in phagocytosis, whereas IL-4 had no effect on phagocytosis in BV-2 cells.

### 2.4. Effects of Different Stimulations on the Energetic Metabolism of Microglia

We assessed the cellular metabolism transition of BV-2 cells under different stimuli, including the level of ATP and lactate, oxygen consumption rate (OCR) and the real-time extracellular acidification rate (ECAR).

**Figure 4:**
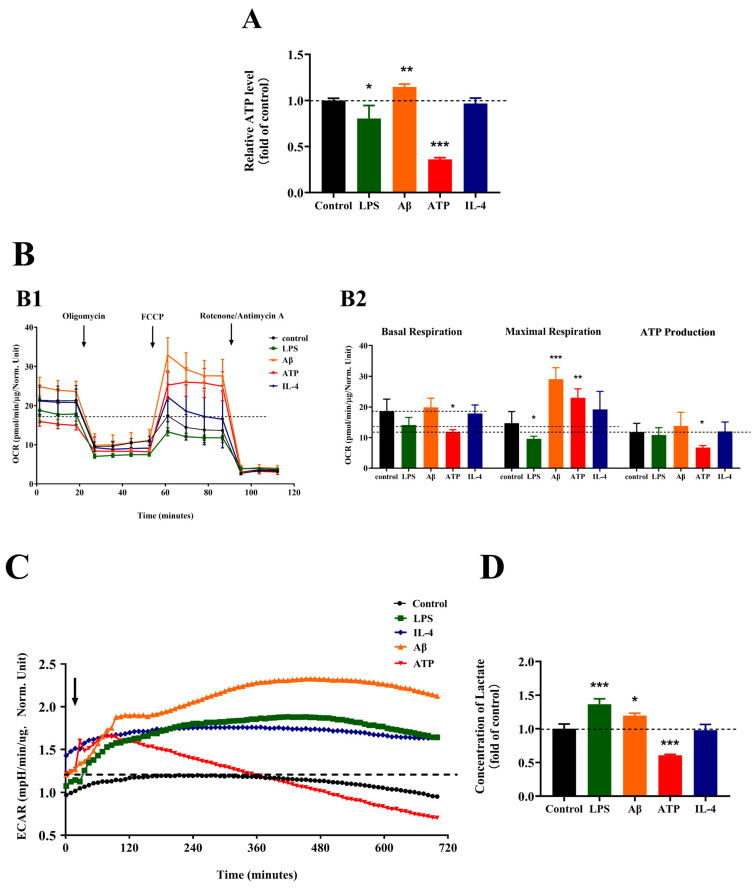
The energetic metabolism of microglia in response to different stimulations. (**A**) The ATP content of BV-2 cells exposed to different stimulations, *n* = 3. (**B**) Oxygen consumption rate (OCR) measurements (B1) and quantitative analysis of basal respiration, maximal respiration and ATP production (B2) of BV-2 cells exposed to different stimulations in the Mito-stress assay, *n* = 4. Oligomycin, an inhibitor of ATP synthase; FCCP (p-Trifluoromethoxy carbonyl cyanide phenylhydrazone), the reversible inhibitor of OXPHOS; Rotenone/Antimycin, the mitochondrial complex I and complex III inhibitor. Mean ± S.D., *n* = 4. (**C**) Extracellular acidification rate (ECAR) measurements of the BV-2 cells exposed to different stimulations, *n* = 4. (**D**) The level of lactate of BV-2 cells exposed to different stimulations, *n* = 3. BV-2 cells were treated with vehicle, 1 µg/mL LPS, 20 µM Aβ, 100 µM ATP or 100 ng/mL IL-4. Mean ± S.D. * *p* < 0.05, ** *p* < 0.01, *** *p* < 0.001, compared with the control groups under the same condition; analyzed by one-way ANOVA with Dunnett’s multiple comparison test for more than two groups. *p* < 0.05 was considered significant.

We first detected the ATP content in different groups and found that the change of ATP content is various, as shown in Figure 4A. The production of ATP in the LPS- and ATP-treated BV-2 cells all decreased (*p* < 0.05 and *p* < 0.001); however, it increased in Aβ-treated BV-2 cells (*p* < 0.01). The group of ATP content with IL-4 treatment was similar to the untreated controls. 

Then, we monitored the OCR of BV-2 cells when treated with different stimuli for 24 h, as shown in Figure 4B. Under LPS stimulation, the OCR decreased; the significantly decreased basal respiration, maximal respiration and ATP production contributed to the OCR decrease (*p* < 0.05). Under ATP stimulation, the OCR decreased, similar to the LPS stimulation, while the basal respiration and ATP production contributed to the OCR change (*p* < 0.05), whereas the maximal respiration significantly increased (*p* < 0.01). These data indicated that the OXPHOS of microglia was suppressed with LPS or ATP treatment. However, the OCR of Aβ-treated BV-2 cells increased; the increased basal respiration, maximal respiration and ATP production led to the OCR increasement (*p* < 0.001), indicating that the Aβ treatment increased the OXPHOS of the microglia. In addition, IL-4 minimally affected the OCR, suggesting that the OXPHOS of microglia were not influenced by IL-4 treatment.

Meanwhile, we monitored the ECAR during 12 h and the lactate content of the BV-2 cells when treated with different stimuli for 24 h, as shown in Figure 4C,D. The ECAR rose sharply after exposure to LPS and reached a high level during 12 h, and the level of lactate was increased (*p* < 0.001) with LPS treatment. In addition, the ECAR rose sharply and reached a high level during 12 h; also, the level of lactate significantly increased (*p* < 0.05) with Aβ treatment. These data demonstrated that the glycolysis of microglia was enhanced under LPS or Aβ exposure. However, the ECAR rose sharply and reached a high level within the initial 2 h, then decreased gradually in the next 10 h, and the level of lactate significantly decreased (*p* < 0.05) after exposure to ATP for 24 h, indicating that ATP treatment decreases the glycolysis of microglia. Consistent with above results of the ATP content and OCR of BV-2 cells with IL-4 treatment, the ECAR and level of lactate were unchanged, indicating that the glycolysis of the microglia was not influenced by IL-4 treatment.

### 2.5. Improving Microglial Cell Type Responses Induced by LPS via Inhibiting Glycolysis

According to the metabolic states of the microglia under PAMP (LPS) stimulation in the above results, glycolysis is required for initiating and maintaining the microglial cell type response. Glucose analog 2-deoxy-D-glucose (2-DG) inhibits the cellular glycolysis pathway. Therefore, we assessed the morphology, fusion rate, cell viability and phagocytosis of LPS-exposed BV-2 cells when pretreated with 2-DG in different doses.

**Figure 5:**
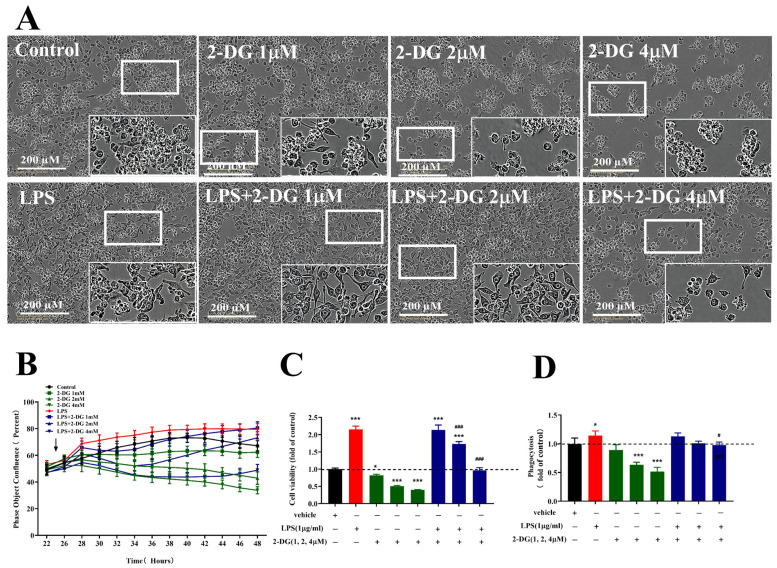
Effects of 2-DG on the morphology (**A**), fusion rate (**B**), cell viability (**C**) and phagocytosis (**D**) in the presence or absence of LPS (1 µg/mL) in microglia. BV-2 cells were pretreated with 2-DG (1, 2, 4 µM) for 2 h and stimulated LPS (1 µg/mL). Data are presented as mean ± S.D., *n* = 4. * *p* < 0.05, *** *p* < 0.001, compared with control group under the same conditions; **^#^** *p* < 0.05, **^###^** *p* < 0.001, compared with LPS 1 µg/mL group under the same condition; analyzed by one-way ANOVA with Dunnett’s multiple comparison test for more than two groups. *p* < 0.05 was considered significant.

We found that the morphology was characterized by the irregular cell shape. The number of cells decreased depending on the concentration of 2-DG (1–4 µM) without LPS treatment, as well as those with LPS treatment, as shown in Figure 5A. Meanwhile, we found that 2-DG reduced the fusion rate of LPS-treated microglia, as shown in Figure 5B. In addition, the cell viability of microglia significantly decreased (*p* < 0.001) depending on the concentration of 1, 2 and 4 µM without LPS treatment, as well as the phagocytosis (*p* < 0.001) in the presence of 2 µM and 4 µM of 2-DG, as shown in Figure 5C,D. The cell viability and phagocytosis of the microglia significantly increased (*p* < 0.001 and *p* < 0.05) upon LPS stimulation, then all of these were reduced (*p* < 0.001 and *p* < 0.05) when pretreated with 2-DG (1–4 µM), as shown in Figure 5C,D. 

### 2.6. Improving Microglial Cell Type Responses Induced by ATP via Promoting Glycolysis

For the effects of DAMPs (Aβ and ATP) on the metabolic states of the microglia, we selected to explore whether the microglial cell type response is improved via promoting glycolysis under ATP stimulation. Fructose-6-phosphate (F6P) is one of the products from the process of glycolysis that promotes the glycolysis. Therefore, we assessed the morphology, fusion rate, cell viability and phagocytosis of ATP-treated BV-2 cells when pretreated with F6P in different doses.

**Figure 6:**
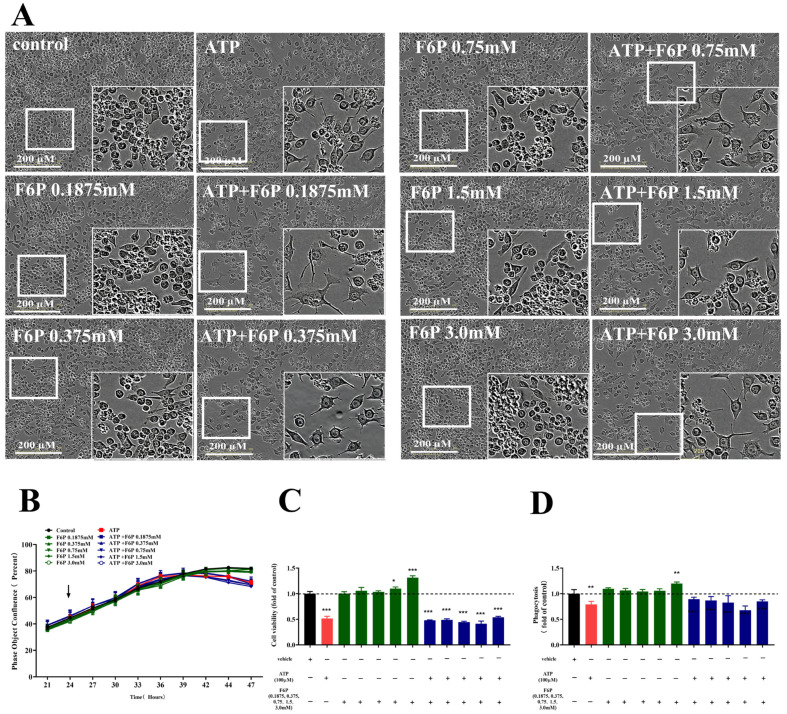
Effects of F6P on the morphology (**A**), fusion rate (**B**), cell viability (**C**) and phagocytosis (**D**) in the presence or absence of ATP in microglia. BV-2 cells were pretreated with F6P (0.187, 0.375, 0.75, 1.5, 3.0 mM) for 2 h and stimulated ATP (100 µM). Data are presented as mean ± S.D., *n* = 3. * *p* < 0.05, ** *p* < 0.01, *** *p* < 0.001, compared with the control group under the same conditions; analyzed by one-way ANOVA with Dunnett’s multiple comparison test for more than two groups. *p* < 0.05 was considered significant.

We found that there was no change in the morphology of the BV-2 cells with F6P treatment (0.1875–3.0 mM) compared with untreated control, as shown in Figure 6A, as well as the fusion rate, as shown in Figure 6B. However, the cell viability and phagocytosis of BV-2 cells significantly increased with F6P treatment at 1.5, 3 mM (*p* < 0.05, *p* < 0.001) or 3.0 mM (*p* < 0.001,), indicating that F6P promoted the glycolysis of the microglia in the quiescent state, as shown in Figure 6C,D. As shown in Figure 5A–D, the ATP activation of BV-2 cells drove the change of morphology with irregular cell bodies and the decreased number of cells, as well as the reduction in the fusion rate, cell viability (*p* < 0.001) and phagocytosis (*p* < 0.01), all of which were not improved by F6P treatment (0.1875–3.0 mM) in ATP-exposed BV-2 cells (Figure 6A–D). These data demonstrate that the cell type response of the microglia induced by ATP was not improved by promoting glycolysis.

## 3. Discussion

The understanding of the metabolic states of microglia in response to different stimulations could be helpful to cover an important strategy associated with AD. Previous evidence has demonstrated a critical role for the energetic metabolism of microglia in immune responses during the development of AD [18,19]. Most previously published studies mainly focus on the metabolic changes of microglia in response to one or some certain kinds of inflammation stimuli, such as LPS, Aꞵ or IL-4. However, there is no systematic study to compare the metabolic changes of microglia under these stimuli, especially ATP stimulation. Our results demonstrated that the microglia showed distinct characteristics under different stimuli exposure, including the cell type response and metabolic status, which enriched and deepened the understanding of the metabolic changes of microglia in response to the inflammatory response in the field of AD. We uncovered that LPS, a proinflammatory PAMP, induced a proinflammatory microglial cell type response with a metabolic shift from OXPHOS to glycolysis, which was similar in several studies [20,21]. Importantly, we found that Aβ and ATP, two kinds of DAMP, induced the opposite changes on cell type response in microglia accompanied by different metabolic states for boosting or enhancing the OXPHOS and glycolysis. Consistent with the effect of IL-4, an anti-inflammatory cytokine, on the microglial cell type response, there also was no effect on the cellular metabolism. Our work demonstrated that in response to different stimuli, the microglia showed a distinct metabolic status that enriched and deepened the understanding of metabolic changes of microglia in response to the inflammatory response in the field of AD. Further, pretreatment with the glycolysis inhibitor (2-DG) improved the proinflammatory cell type response induced by LPS, a kind of PAMPs, in microglia. However, pretreatment with the glycolysis accelerator (F6P) showed no prominent effects on the cell type response induced by ATP, a kind of DAMPs, in microglia. Therefore, targeting the cellular metabolism may provide a powerful approach to tune the microglia immune responses.

As a typical proinflammatory PAMP, LPS induces neuroinflammation and contributes to the changes in the microglial cell type response, including the morphology, fusion rate, cell viability and phagocytosis [22,23]. Previous work with BV-2 cells demonstrated that LPS causes an inhibition of OXPHOS and an induction of glycolysis [11,17]. The accepted view is that proinflammatory PAMPs switch a glycolytic phenotype quickly in microglia, and this rapid change is considered to support the immune function of microglia [11,17,20,24,25]. During this process, the ATP level in microglia was decreased, although the switch to aerobic glycolysis indicates that it is not enough to provide enough energy to support microglial functions when the pathway of OXPHOS was shut down. In addition, the proinflammatory stimulation (LPS) prominently altered the expression of the genes of the microglia involved in glucose metabolism, with RNA transcriptional profiling to support the metabolic change [24]. Upon LPS stimulation, the microglia further upregulate glucose transporter 1 (GLUT1) to facilitate the glucose uptake and promote anaerobic glycolysis [17]. Therefore, glycolysis is vital for the inflammatory response in LPS-activated microglia. We observed that blocking glucose metabolism by 2-DG robustly suppressed the increasement of LPS-treated microglia on the cell type response, in accordance with the suppressed neuroinflammation, reduced microglia activation and attenuated Aβ pathology after 2-DG treatment in cell models of AD [21,25,26]. LPS enhances the production of proinflammatory cytokines in the microglia, moreover, blocking the glucose metabolism by 2-DG suppressed the production of proinflammation cytokines [24,25]. In addition, in our previous study we also found similar results that microglia secreted cytokines, including TNF-α, IL-6 and IL-10, after LPS stimulation. These data indicate that targeting the energetic metabolism, particularly glycolysis, may be a potential approach to alleviate microglia-mediated neuroinflammation in AD [18,20,24,27].

Notably, we found that markedly different metabolic states are induced by DAMPs, which are distinct from the metabolic states induced by PAMPs. We observed that the DAMP Aβ caused both OXPHOS and glycolytic induction, whereas the DAMP ATP caused both OXPHOS and glycolysis inhibition in the microglia. The result from RNA transcriptional profiling showed a higher expression of the genes associated with glycolysis in the microglia [28]; also, the result of Western blot analysis showed that Aβ induced high levels of glycolysis intermediates, including glucose-6-P, fructose-6-P, fructose-1,6-dP, glyceraldehyde-3-P, 3-phosphoglyceric acid, phosphoenolpyruvate, pyruvic acid and lactic acid, in microglia [29], indicating a shift toward a glycolytic metabolism. The microglia express a wide range of receptors that play an important role in multiple aspects of DAMP-induced effects in the microglia. For example, TLR2 receptors respond to Aβ, which drives the enhancement of OXPHOS and glycolysis, which is similar to the metabolic changes induced by the TLR2 ligand Pam3CysSK4 (P3C) in human monocytes [30], as well as the observation of the metabolic change induced by Aβ in our study. In addition, microglia express P2Y12 receptors to recognize ATP. The microglia involved in neuroinflammation have cell type response changes in response to ATP in the central nervous system, via the P2Y12 receptors [31,32,33]. However, we know little about the metabolic states of the microglia induced by ATP. So far, it just has been reported that ATPγS, a nonhydrolyzable ATP analogue, enhanced the OXPHOS and glycolytic capacity of rat primary microglia [22]. Here, the ATP level, glycolysis and OXPHOS were reduced by 100 µM ATP, and the fusion rate, cell viability and phagocytosis were decreased upon ATP stimulation. It is indicated that the energetic metabolism was weakened, leading to damaged effects on the microglial cell type response. For the importance of the energetic metabolism of DAMP-treated microglia, we explored whether it is a potential therapeutic approach for regulating the metabolism to alleviate the neuroinflammation mediated by microglia under ATP stimulation. The results showed that the microglial cell type response was not improved by promoting glycolysis, although which were enhanced at quiescent state. Maybe the microglial cell type response under ATP exposure can be improved by regulating OXPHOS. In conclusion, it may be a potential method for regulating the cellular metabolism to reduce injury caused by DAMP in microglia.

Although it has been reported that the microglia depend on OXPHOS when subject to IL-4, an anti-inflammatory immunomodulatory cytokine, according to the previous study [34,35,36], there is uncertainty about the metabolic states of microglia upon IL-4 stimulation. It has been reported that the metabolic states of IL-4-treated rat primary microglia were unchanged on both OXPHOS and glycolysis [24], as was the phenomenon we observed here in BV-2 cells. The result of the RNA transcriptional profiling showed that IL-4 minimally affected the expression of genes in the glucose metabolism of microglia [24]. All data support our work on the effect of IL-4 in microglia.

In summary, we have observed the changes of the microglial cell type response induced by PAMPs, DAMPs and anti-inflammatory immunomodulatory cytokines accompanied by various metabolic states. In addition, we performed a similar study in the microglia derived from human-induced pluripotent stem cells (hiPSCs), which have not yet been published, to expand our understanding of the microglial metabolism in AD. In addition, we can regulate the microglial metabolism to alleviate the neuroinflammation mediated by the microglia in AD.

## 4. Materials and Methods

### 4.1. Cell Culture and Drug Treatment

BV-2 cells (the Institute of Basic Medicine Chinese Academy of Medical Sciences, Beijing, China) were maintained in high glucose DMEM (Sigma, St. Louis, MO, USA, Catalog No. D6429), which contained 0.45% glucose supplemented with 10% FBS (Sigma, St. Louis, MO, USA, Catalog No. 12103C) at 37 °C, in a humidified atmosphere of 5% CO_2_ in air. The BV-2 cells were plated in culture plates at the same density with 1 × 10^4^ cells per well for the different stimuli.

For different stimulation treatments, all cells were divided into the following groups: a control group; an LPS (Merck, Darmstadt, Germany, Catalog No. L4391) group; an IL-4 (Merck, Darmstadt, Germany, Catalog No. SRP3211) group; an Aβ (Aladdin, shanghai, China, Catalog No. A118755) group; and an ATP (Merck, Catalog No. A1852) group. The cells were stimulated with LPS, IL-4, Aβ or ATP for 24 h, then stored for later tests.

For the drug treatment, all cells were divided into the following groups: a control group; a 2-DG (Solarbio Life Science, Beijing, China, Catalog No. 154-17-6) group (1, 2, 4 mM) or a F6P (Solarbio Life Science, Beijing, China, Catalog No. D8010) group (0.1875, 0.375, 0.75, 1.5, 3.0 mM); an ATP group (100 µM); and a 2-DG + ATP or F6P + ATP group. The cells were incubated with 2-DG or F6P for 2 h, and subsequently stimulated with LPS or ATP for 24 h, then stored for later tests.

### 4.2. Cell Viability Assay

To assess the viability of the BV-2 cells, the culture medium was discarded, then 100 µL fresh culture medium containing 10 µL CCK-8 reagent (DOJINDO, Rockville, MD, USA, Catalog No. CK04) per well was added after being stimulated by the different stimulations. The cells continued to be cultured for 1 h. Finally, the optical density (OD) absorbance was measured at a wavelength of 450 nm. The cell survival rate was expressed as A450 nm.

### 4.3. Phagocytosis Assay

To assess the phagocytosis of the BV-2 cells, the culture medium was discarded, then 100 µL PBS containing 0.05% neutral red (Sinopharm Chemical Reagent Co., Ltd., Shanghai, China, Catalog No. 71028144) was added per well after being stimulated by the different stimulations. The cells continued to be cultured for 30 min at 37 °C. Then, the cells were washed thrice with PBS, 100 µL of cell lysate (V (glacial acetic acid):V (ethanol) = l:1) was added to each well and the plates were incubated at 4 °C for 2–3 h. Finally, the optical density (OD) absorbance was measured at a wavelength of 540 nm. The phagocytosis of BV-2 was expressed as A540 nm.

### 4.4. Measurement of the ATP Content

To assess the ATP content in BV-2 cells, 100 µL of lysis buffer containing luciferase reagents (Promega, Madison, WI, USA, Catalog No. G7570), the same volume as the cultured medium, was added to the cells per well and incubated for 10 min. Finally, the luminescent signal was measured in a microplate reader and the ATP content was normalized to the protein concentration.

### 4.5. Measurement of Lactate

To assess the levels of lactate in BV-2 cells, the lactate secreted into the cultured medium was quantified using a Lactate Assay Kit-WST kit (DOJINDO, Rockville, MD, USA, Catalog No. L256) according to the manufacturer’s instructions. Finally, the levels of lactate were measured at a wavelength of 450 nm. The levels of lactate were expressed as the concentration of lactate.

### 4.6. Metabolic Extracellular Flux Analysis

The bioenergetic properties of the BV-2 cells were determined under different stimulation exposures using the XF-24 Seahorse extracellular flux analyzer (Seahorse Bioscience, Santa Clara, CA, USA). It measures the real-time changes in extracellular acidification rate (ECAR) and the oxygen consumption rate (OCR) that are indicative of glycolysis and mitochondrial respiration, respectively. In short, the microglia were plated on XF-24 cell culture plates in the presence or absence of LPS, IL-4, Aβ or ATP. At the indicated time points, the microglia were washed and analyzed in the XF Running buffer (XF assay medium contained 10 mM glucose, 1 mM pyruvate sodium, 2 mM L-glutamine) for the OCR or ECAR measurements. The OCR measurements were obtained in real-time with no drug treatment (basal conditions) and with the sequential treatment of different drugs: 2 μM oligomycin, 1 μM FCCP, 0.5 μM rotenone plus 0.5 μM antimycin A. For the ECAR measurements, vehicle, LPS, IL-4, Aβ and ATP were injected into cells 30 min after loading the plate into the XF analyzer and allowing it to stabilize, then ECAR was monitored every 6 min for 12 h. Upon completion of the measurements, the number of cells within the plate was determined by the quantitative protein. Data were obtained as the pH change and picomoles of O_2_ consumption per min in the media and normalized to the cell number.

### 4.7. Statistical Analyses

All data were expressed as mean ± SD. All the statistical analyses were performed using GraphPad Prism version 8.0 software. The significance of differences was assessed by one-way analysis of variance (ANOVA) followed by Dunnett’s multiple comparison test for more than two groups. The significant threshold was set at *p* < 0.05.

## Data Availability

The original contributions presented in the study are included in the article, further inquiries can be directed to the corresponding author.
